# A 25-year retrospective study of *Chlamydia psittaci* in association with equine reproductive loss in Australia

**DOI:** 10.1099/jmm.0.001284

**Published:** 2020-12-01

**Authors:** Rumana Akter, Fiona M. Sansom, Charles M. El-Hage, James R. Gilkerson, Alistair R. Legione, Joanne M. Devlin

**Affiliations:** ^1^​ Asia Pacific Centre for Animal Health, The Melbourne Veterinary School, The University of Melbourne, Parkville, Victoria 3010, Australia; ^2^​ Department of Medicine (Royal Melbourne Hospital), The University of Melbourne, Parkville, Victoria 3010, Australia

**Keywords:** abortion, Australia, *Chlamydia psittaci*, equine, horse, zoonosis

## Abstract

**Introduction:**

*
Chlamydia psittaci* is primarily a pathogen of birds but can also cause disease in other species. Equine reproductive loss caused by *C. psittaci* has recently been identified in Australia where cases of human disease were also reported in individuals exposed to foetal membranes from an ill neonatal foal in New South Wales.

**Hypothesis/Gap Statement:**

The prevalence of *C. psittaci* in association with equine reproductive over time and in different regions of Australia is not known.

**Aim:**

This study was conducted to detect *C. psittaci* in equine abortion cases in Australia using archived samples spanning 25 years.

**Methodology:**

We tested for *C. psittaci* in 600 equine abortion cases reported in Australia between 1994 to 2019 using a *Chlamydiaceae* real-time quantitative PCR assay targeting the 16S rRNA gene followed by high-resolution melt curve analysis. Genotyping and phylogenetic analysis was performed on positive samples.

**Results:**

The overall prevalence of *C. psittaci* in material from equine abortion cases was 6.5 %. *C. psittaci *-positive cases were detected in most years that were represented in this study and occurred in Victoria (prevalence of 7.6 %), New South Wales (prevalence of 3.9 %) and South Australia (prevalence of 15.4 %). Genotyping and phylogenetic analysis showed that the *C. psittaci* detected in the equine abortion cases clustered with the parrot-associated 6BC clade (genotype A/ST24), indicating that infection of horses may be due to spillover from native Australian parrots.

**Conclusion:**

This work suggests that *C. psittaci* has been a signiﬁcant agent of equine abortion in Australia for several decades and underscores the importance of taking appropriate protective measures to avoid infection when handling equine aborted material.

## Introduction

*Chlamydia psittaci* is an obligate intracellular Gram-negative bacterium that belongs to the family *Chlamydiaceae* [1]. *C. psittaci* infection causes a disease known as psittacosis. *C. psittaci* is mainly transmitted by inhalation and the severity of the disease can vary from a subclinical infection or mild respiratory disease to severe pneumonia and systemic psittacosis [2, 3]. Birds are the primary reservoir host, but the organism has a wide host range, including humans and other mammals [1]. Human psittacosis is an important public health concern. Transmission of infection from birds to humans is frequently reported [4–7]. In humans, infection can cause flu-like symptoms, which are often associated with severe respiratory illness and multi-organ involvement that can lead to significant morbidity and mortality [8]. Transmission of *C. psittaci* to humans is usually due to interactions with infected birds or bird excreta [3, 9–12].

*C. psittaci* has been detected in many species, including cattle, buffalo, goats, sheep and horses [13–16]. Infection in livestock can be associated with respiratory, intestinal or arthritic diseases and also abortion [17]. Until recently, *C. psittaci* infection has frequently been overlooked amongst the differential diagnoses of equine reproductive loss, and limited epidemiological studies have been conducted. This is despite horses being occasional hosts of *C. psittaci *, where infection may result in respiratory disease and foetal abortion [13, 17, 18]. Recently *C. psittaci* infection in horses has been investigated in more detail and has been detected in association with equine abortion cases in Germany [19] and in Hungary, where *C. psittaci* was detected in 11/77 cases (14.3 %) of equine reproductive loss [17, 20]. In Australia, *C. psittaci* was responsible for an outbreak of disease in five staff and students at a veterinary school following contact with foetal membranes from a weak foal in 2014 [12]. This prompted a number of related studies [12, 21, 22] that demonstrated *C. psittaci* in 21 % of cases of equine reproductive loss in New South Wales (NSW), Australia [21]. Most recently *C. psittaci* has also been identified as the suspected cause of equine reproductive loss in three cases in Victoria (VIC), Australia [23]. Sequence analysis of the outer-membrane protein A (*omp*A) as well as multi-locus sequence typing (MLST) revealed that the detected *C. psittaci* clustered with the avian 6BC clade [17, 24]. The 6BC clade is highly virulent and can readily infect humans to cause serious disease [25–27].

The significance of *C. psittaci* infection as a cause of equine abortion in other areas of Australia, or over time, is currently unknown. To investigate this, we conducted a retrospective study to detect *C. psittaci* in association with the equine abortion cases in Australia using 25 years of archived samples.

## Methods

### Sample collection

Multiple tissue samples from equine abortion cases from 1994 to 2019 were submitted to our diagnostic laboratories within the Centre for Equine Infectious Disease (CEID), and the Asia-Pacific Centre for Animal Health (APCAH) in Melbourne, Australia, or were supplied to our laboratories after initial submission to diagnostic laboratories at Agriculture Victoria. The samples were collected and submitted to diagnostic laboratories for purposes unrelated to this study. Excess material (beyond the amount that was required for initial diagnostic testing) from each sample was stored in laboratory archives and were used in this study. Based on the availability of tissues, a total of 600 cases were selected from the archive with a sample of lung, spleen, thymus and placenta collected from each case. Of these 600 cases, 395 were from VIC, 182 were from NSW, 4 were from Queensland (QLD), 4 from Western Australia (WA), 1 was from Tasmania (TAS), 13 were from South Australia (SA) and 1 was from the Northern Territory (NT). The tissues had been stored at −80 °C in 1.5 ml tubes after submission. Selected samples were thawed, and a plastic-shafted rayon tipped swab (Copan Italia) was used to sample each tissue. Different tissue swabs from each foetus were combined in 500 µl phosphate-buffered saline (PBS) and pooled swabs from each individual foetus were stored at −80 °C until DNA extraction.

### DNA extraction

Each tube of swab/PBS solution was vortexed for approximately 5 s before a 200 µl aliquot was removed for DNA extraction. DNA was extracted from the PBS solution by a Kingfisher robot with a MagMAX Core Nucleic Acid Purification kit (Thermo Fisher Scientific) according to the manufacturer’s instructions. Plasmids (pGEM-T, Promega) containing the 16S rRNA gene of *C. psittaci *, *Chlamydia pecorum* and *Chlamydia pneumoniae* were used in positive extraction control reactions and PBS was used as a negative extraction control. Extracted DNA was eluted in 90 µl of elution buffer and stored at −80 °C for further use. These extraction methods were followed for the extraction of 483 swab/PBS pools encompassing samples collected between 1994 to 2004. The remaining 117 samples submitted between 2010 to 2019 had been pre-extracted from tissue swabs (pooled for each individual foetus/case) for routine diagnostic testing and stored prior to this study.

### qPCR to detect *Chlamydiaceae*

Swabs of foetal tissues (pooled for each individual foetus/case) were screened using a *Chlamydiaceae* real-time quantitative PCR/high-resolution melt curve analysis (qPCR-HRM) assay targeting a 460 bp region of the 16S rRNA gene of *Chlamydiaceae* as described previously [28]. Positive control samples used DNA template extracted from the pGEM-T cloned target sequences of *C. psittaci *, *C. pecorum* and *C. pneumoniae *. Milli-Q filtered water was used as negative control. The curves produced from control samples of *Chlamydia* DNA were used to compare the profile of the melting curve for each clinical sample. Positive detection by fluorescence were confirmed by comparing melt curves to positive control samples and the samples were considered to be positive if the *C*_t_ value was <35 and the profile of the melt curve was consistent with those generated from positive control samples. The limit of the assay for the detection of each plasmid was determined by testing 10-fold serial dilutions from 10^8^ to 10^1^ copies of the targeted plasmid in triplicate. A Qubit 3.0 fluorometer (Invitrogen) was used to calculate the copy numbers of plasmid.

### Genotyping of *C. psittaci* using the *ompA* gene

The *C. psittaci ompA* gene was amplified for genotyping as described previously [29]. All PCRs were carried out in a T100 Bio-Rad thermal cycler. The amplified PCR products were visualized by UV transillumination using Bio-Rad Image Lab software after agarose gel electrophoresis of 10 µl aliquots through a 2 % agarose gel containing SYBR Safe DNA Gel Stain (Thermo Fisher Scientific) and run in 45 mM Tris-borate/1 mM EDTA buffer at 90 V for 40 min. Hyper ladder 1 kb (Bioline) was used for the estimation of amplicon size. The expected size of the amplicon was 1200 bp.

### MLST

MLST was performed on 39 *C *. *psittaci *-positive samples according to the scheme previously developed by Pannekoek *et al*. [30]. Fragments of seven housekeeping genes (*gatA*, *oppA*, *hflX*, *gidA*, *enoA*, *hemN* and *fumC*) of *Chlamydiales* were amplified (381–635 bp in length) and sequenced using the oligonucleotide primers previously published on the *Chlamydiales* MLST website (https://pubmlst.org/chlamydiales/info/MLST_primers_2012.pdf) [31]. All PCR was carried out in a T100 Bio-Rad thermal cycler as described previously [32] and visualized as described for *ompA*.

### Sequencing and phylogenetic analysis

PCR products were purified from the PCR reaction mixtures using the QIAquick Gel Extraction kit (Qiagen) according to the manufacturer’s instructions and eluted in 30 µl elution buffer (10 mM Tris/Cl, pH 8.5). The quantity of purified DNA was estimated using a spectrophotometer (NanoDrop Technologies) and sequenced using Big Dye Terminator (BDT) v3.1 (Life Technologies) according to the manufacturer’s instructions. The purified products were sent to the Australian Genome Research Facility (AGRF) for capillary separation. Geneious bioinformatics software version 11.1.4 (Biomatters) was used to trim, manually curate and align all obtained sequences [33]. Nucleotide sequences were compared with publicly available sequences in the GenBank database [34] using the National Center for Biotechnology Information (NCBI) Nucleotide Basic Local Alignment Search Tool (blastn) online algorithm [35]. *C. psittaci ompA* sequences were aligned with previously published sequences (accession nos: AY762610.1, AF269260.1, NZ_LZRX01000001.1, NZ_LZRF01000001.1, NZ_LZRE01000001.1, KF770962.1, AY762613.1, AY762611.1, PJPZ01000004.1, M73035.1, CP002586.1, CP003795.1, CP003796.1, CP025423.1, PJPY01000001.1, NZ_LZRY00000000.1, NZ_LZRZ00000000.1 and AF269282.1). MLST of *C. psittaci* sequences for each locus were queried against the online *Chlamydiales* MLST database (https://pubmlst.org/chlamydiales) (Table S1, available in the online version of this article) to determine allelic designation, and the sequence types (STs) were identified using the subsequent allelic profile. Previously published sequences were retrieved from the MLST database to use as references [31, 36]. The alignment of *ompA* and concatenated MLST fragments were used to generate a phylogenetic tree. A PhyML phylogenetic tree with 500 bootstraps [37] and the GTR+I model was generated using multiple sequence MAFFT alignment of positive strains, implemented in Geneious software version 11.1.4.

### Statistical analysis

The statistical significance of the prevalence of *C. psittaci* infection among different states was tested using Fisher’s exact test in IBM SPSS Statistics 25. The load of *C. psittaci* infection in the positive samples was compared between samples originating from different states using a Kruskal–Wallis test in GraphPad Prism 8. *P* values <0.05 were considered significant.

## Results

### Detection of *C. psittaci* in equine abortion cases from Australia

**Fig. 1.**
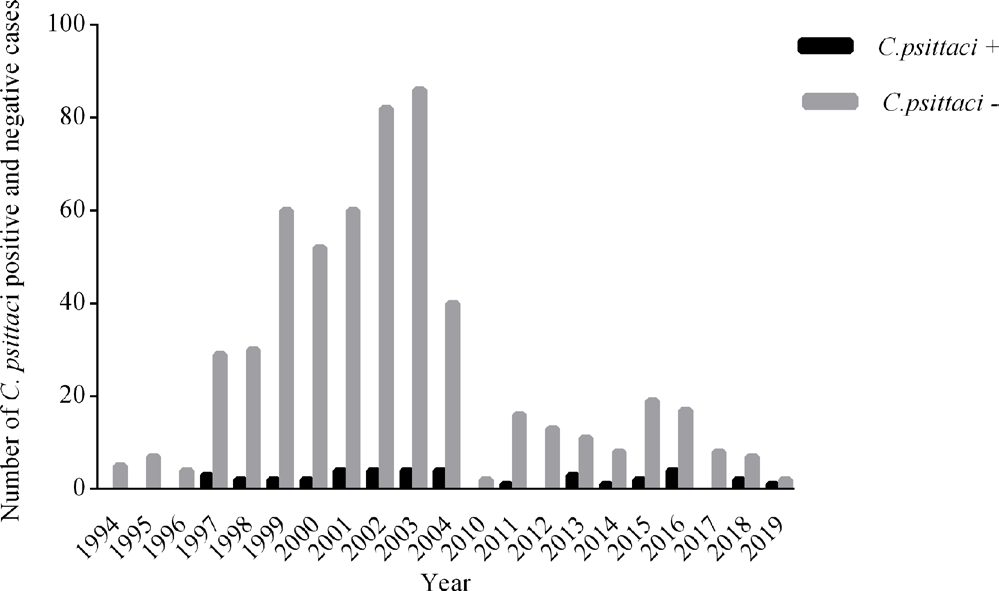
Distribution of *Chlamydia psittaci* positive and negative cases between 1994–2019.

**Fig. 2.**
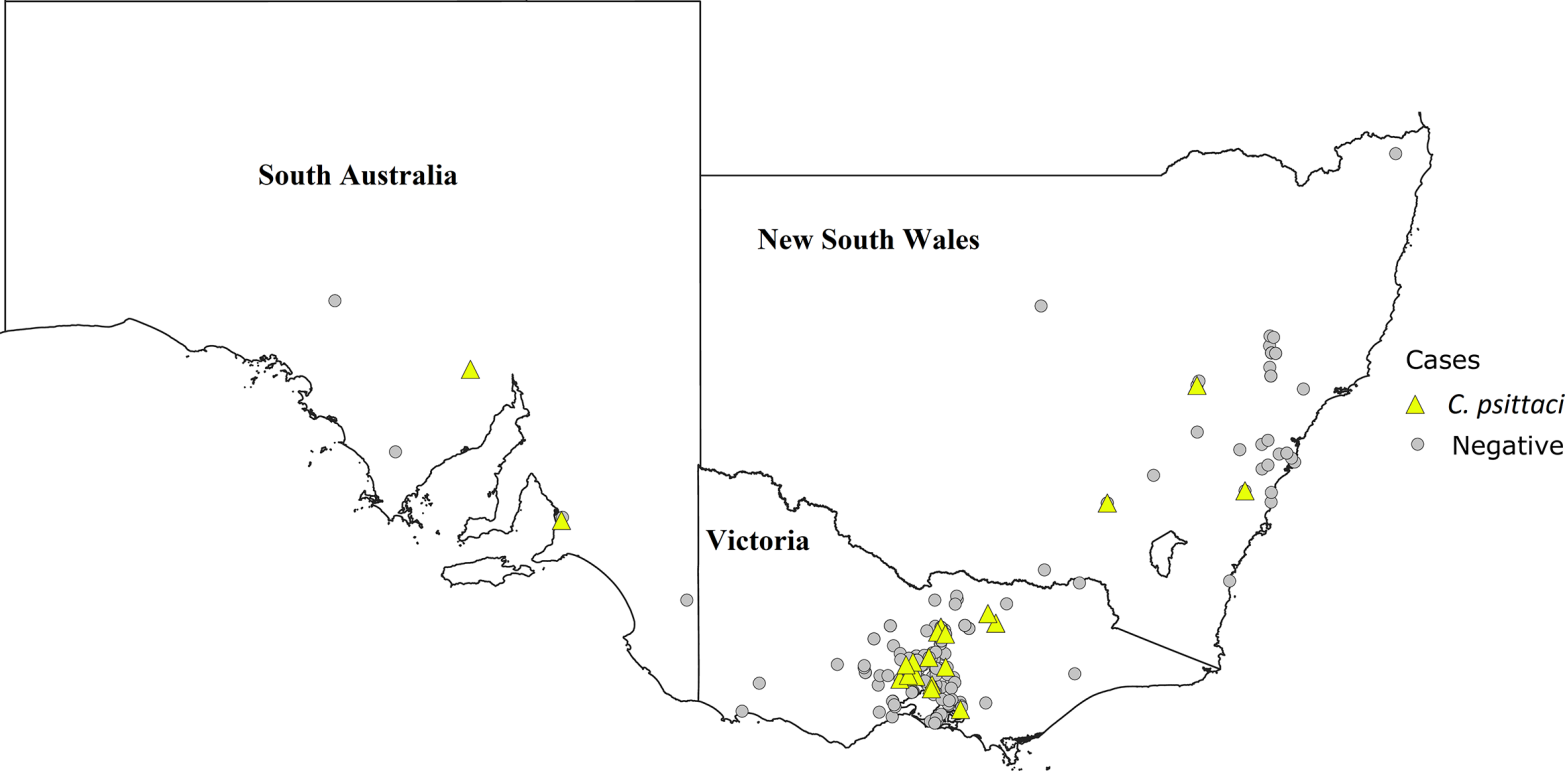
Map of the states of SA, VIC and NSW, Australia, showing the locations of equine abortion cases. Here, *Chlamydia psittaci* positive and negative cases are represented by yellow triangles and grey circles, respectively. Multiple cases originating from the same location are represented only once. A positive symbol is shown at a location if any positive cases were detected amongst the multiple cases originating from that location.

We screened 600 cases of equine abortion from 1994 to 2019 using qPCR-HRM. Of the 600 cases, 39 cases were positive for *C. psittaci *, giving a prevalence of 6.5 % (95 % CI: 4.8–8.8 %). *C. psittaci* were detected in abortion cases in almost every year (Fig. 1). Most of the samples in this study originated from VIC and NSW and the prevalence of *C. psittaci* in these states was 7.6 % (30/395, 95 % CI: 5.4–10.6 %) and 3.9 % (7/182, 95 % CI: 1.9–7.7 %), respectively. *C. psittaci* was also detected in 2/13 samples that were submitted from SA(prevalence of 15.4 %, CI: 4.3–42.2 %). The distribution of positive and negative cases in NSW, VIC and SA is shown in Fig. 2. The numbers of positive and negative cases are shown in Table 1. *C. psittaci* was not detected in the states of QLD, TAS, WA or the NT. There was no significant difference in the prevalence of C. *psittaci* infection in the different Australian states.

**Table 1.** Prevalence of infection and *Chlamydia psittaci* loads in samples originating from different Australian states

Australian state or territory	* C. psittaci *-positive cases/*C.psittaci*-negative cases	% of positive cases (95 % confidence interval)	Median load (genome copiesµl^−1^ extract)	Range (genome copies µl^−1^ of extract)
Victoria	30/365	7.6 (5.4–10.6)	1.41×10^3^	1.28×10^1^–1.19×10^7^
New South Wales	7/175	3.9 (1.9–7.7)	5.70×10^3^	3.10×10^1^–6.92×10^6^
South Australia	2/11	15.4 (4.3–42.2)	8.46×10^4^	1.69×10^2^–1.69×10^5^
Queensland	0/4	0 (0.0–48.9)	na	na
Tasmania	0/1	0 (0.0–79.4)	na	na
Northern Territory	0/1	0 (0.0–79.4)	na	na
Western Australia	0/4	0 (0.0–48.9)	na	na
All	39/561	6.5 (4.7–8.8)	1.16×10^3^	1.28×10^1^–1.19×10^7^

### *C. psittaci* load

Genome copy numbers of *C. psittaci* in positive samples (*n*=39) were quantified by 16S rRNA gene PCR. The median (range) of the *C. psittaci* load was 1.16×10^3^ (1.28×10^1^–1.19×10^7^) genome copy numbers µl^−1^ of extract. *C. psittaci* loads for samples originating in the different states is shown in Table 1. There was no significant difference in the load of C. *psittaci* infection in samples originating from the different Australian states.

### Genotyping of *C. psittaci*

**Fig. 3.**
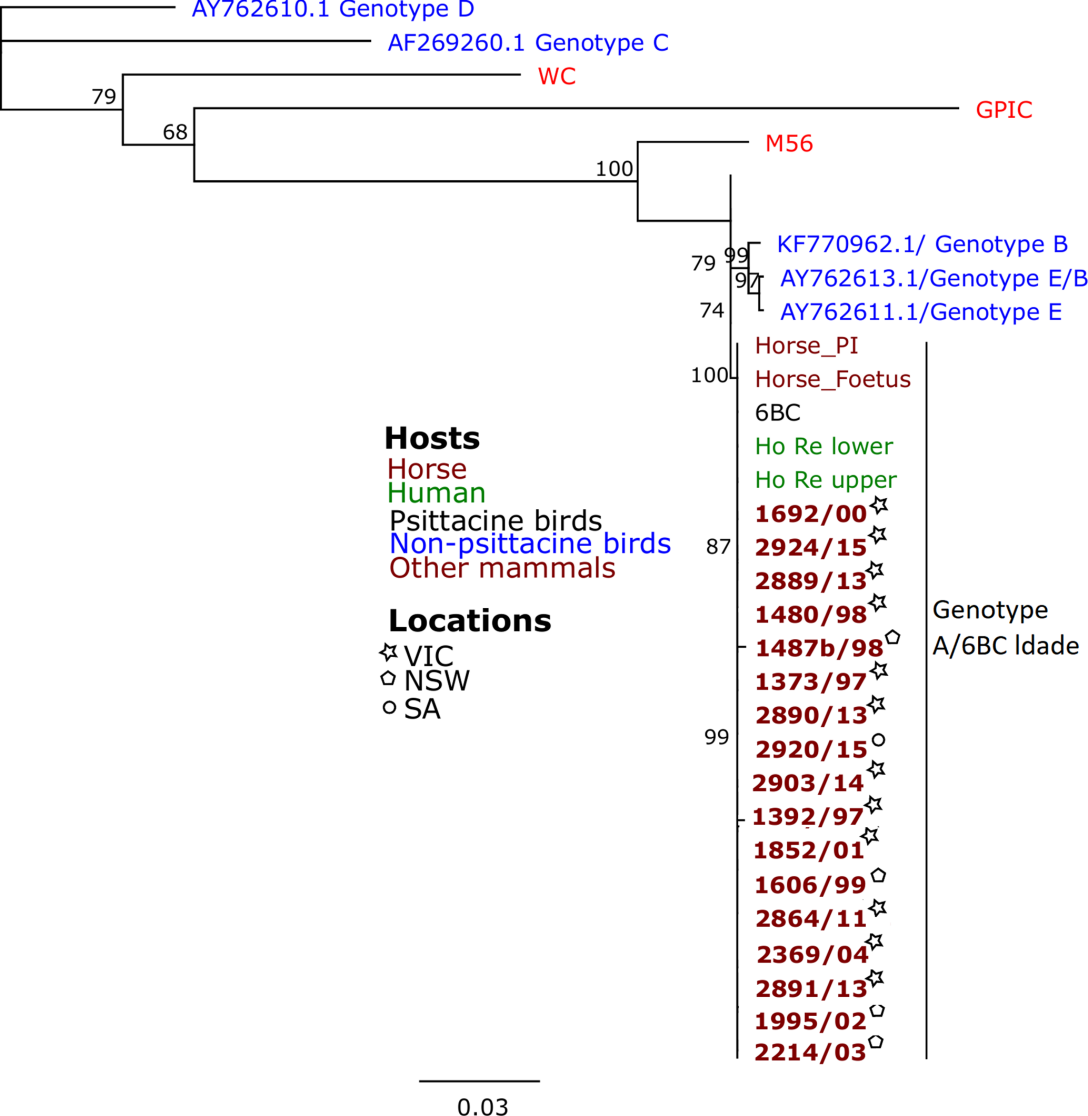
Phylogeny of *Chlamydia psittaci* detected in this study along with *C. psittaci* from other Australian and global studies. Mid-point rooted PhyML phylogenetic tree with 500 bootstraps using the alignment of the *ompA* gene fragment (991 bp) sequences of Australian *C. psittaci* samples. *C. psittaci* sequences from this study are in bold. Host and locations of isolates are denoted by different colours and symbols. Bootstrap values greater than 60 are shown on the tree nodes.

**Fig. 4.**
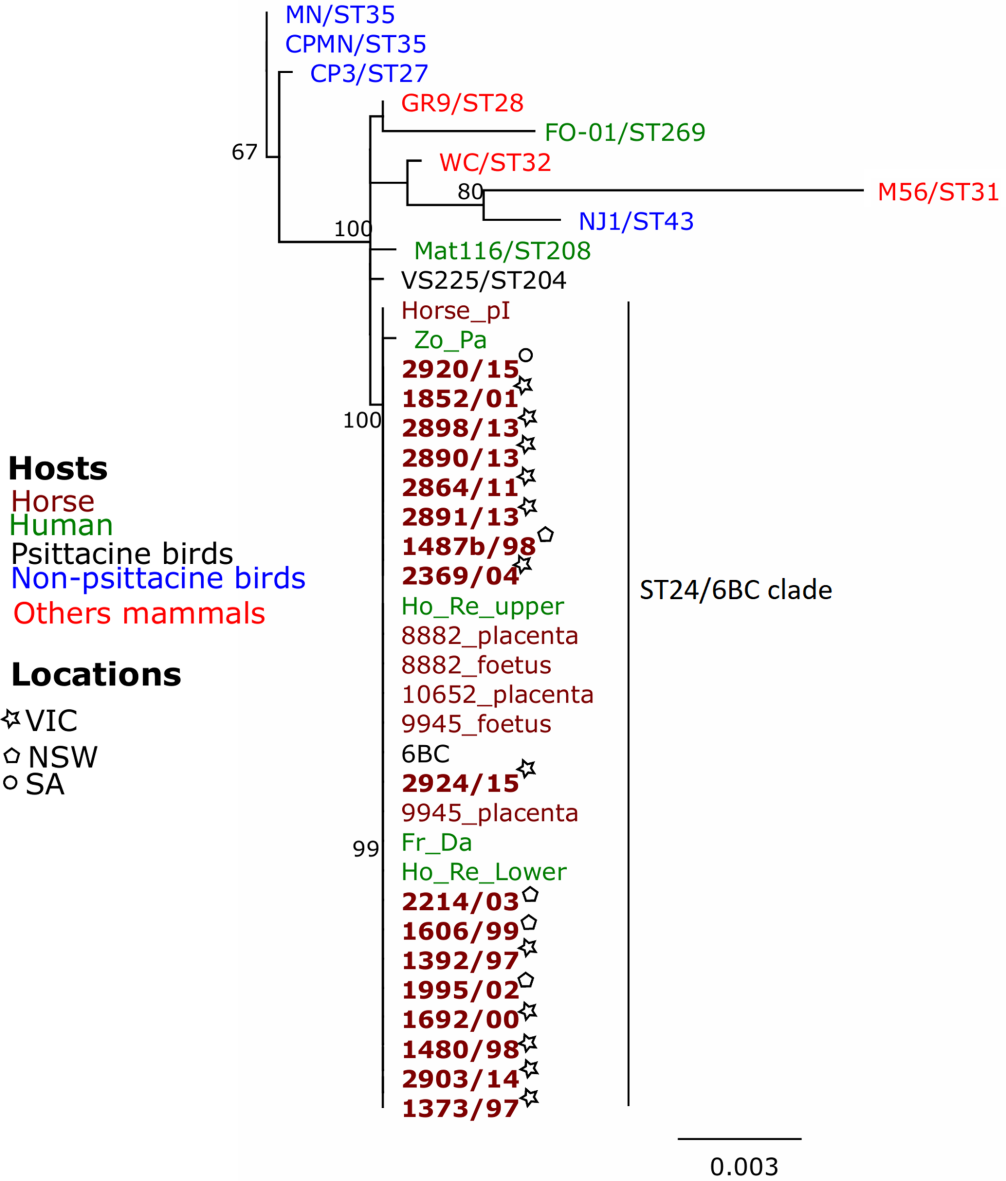
Mid-point rooted PhyML phylogenetic tree with 500 bootstraps using the alignment of the concatenated MLST fragment sequences (3098 bp) of Australian *Chlamydia psittaci* strain samples. *C. psittaci* sequences from this study are in bold. Host and locations of isolates are denoted by different colours and symbols. Bootstrap values greater than 60 are shown on the tree nodes.

We successfully genotyped 17 (out of 39) positive samples. The *ompA* sequences were highly homologous and shared 99.2–100 % similarity. The *ompA* phylogeny analysis revealed that all the samples sequenced in this study clustered with previously detected equine samples and with the highly virulent clade 6BC isolates, belonging to genotype A (Fig. 3) [24]. Our samples also clustered with *C. psittaci* previously detected in Australian parrots and humans. MLST analysis applied to the *C. psittaci* positive samples showed that they all belonged to ST24 (Table S2) and clustered with *C. psittaci* previously detected in horses, Australian parrots and humans (Fig. 4) [24, 26].

## Discussion

Our study showed that the overall prevalence of *C. psittaci* in the equine abortion samples was 6.5 %. *C. psittaci* DNA was detected in most of the years represented in this study, with the earliest postitive sample collected in 1997. These results indicate that *C. psittaci* is not an emerging cause of equine infectious abortion in Australia, but rather an underdiagnosed infection that has been present for more than two decades. Most of the positive cases were from VIC and NSW, reflecting the large number of samples from these states, but *C. psittaci* was also detected in two samples from SA, indicating that equine abortion due to *C. psittaci* is geographically widespread.

*C. psittaci* was not detected in the cases from QLD, NT, TAS and WA. Limited samples were tested from these states or territories and this may explain why *C. psittaci* was not detected. *C. psittaci* is known to be present in other species (particularly birds and humans) in all Australian states and territories [32, 38–40], but has only previously been implicated as a cause of equine abortion in NSW and VIC [21, 23]. The C. *psittaci *-negative samples from QLD, NT, TAS and WA were all collected in years during which *C. psittaci* was detected in VIC and/or NSW, but there are large geographical distances and climatic differences between regions, In the future, including a larger number of samples from all over Australia would help to better understand *C. psittaci* infection in Australian horses.

The prevalence of *C. psittaci* in this study (6.5 %) was lower than that reported in a previous study of equine reproductive loss in NSW in 2016 (21.1 %) [21]. The reasons behind this are unclear, but it is likely that the prevalence of *C. psittaci* in equine abortion cases varies from year to year, possibly with environmental conditions [10, 11, 38]. Variability between years was detected in our current study (Fig. 1). A range of different *C. psittaci* prevalence rates, ranging from 14.3–27.1 %, have been reported in studies of equine reproductive loss in Europe[41] . In other countries, other species of *Chlamydia,* including *C. abortus* and *C. suis *, have been detected by qPCR [42]. The qPCR-HRM system used in this current study is able to detect and differentiate species within the family *Chlamydiaceae* [28], but no other species were detected in the samples, which is consistent with other Australian studies. Importantly, *C. abortus* is exotic to Australia and was not detected [21,43,44].

In a previous study of equine abortion in Australia, median *C. psittaci* loads were 1.07×10^5^ genome copies µl^−1^ of extract in placental tissue and 8.31×10^2^ genome copies µl^−1^ of extract in foetal tissues [21]. In our study we tested a combined mixture of placental and foetal tissues and the median load was 1.16×10^3^ genome copies µl^−1^ of extract. Detection of *C. psittaci* alone does not necessarily indicate that it was the cause of the abortion event. Ideally, histopathological examination of the aborted material would be conducted, but these data were not available for the samples used in the current study. Future prospective studies would ideally combine histopathological and molecular approaches to confirm the involvement of *C. psittaci* in equine abortion cases, as has been performed in other studies [21].

*C. psittaci* has previously been detected in equine foetal liver, thymus, lung, heart and spleen, as well as in the equine placenta [17, 21]. Histological lesions, including mild interstitial pneumonia, thrombi and perivasculitis in foetal lungs, have been reported, as well as multiple necrotic foci and mild lympho-histiocytic infiltration in foetal liver [17, 21]. *C. psittaci* has also been associated with mild lympho-histiocytic placentitis in horses. In some equine abortion cases *C. psittaci* has been detected without any gross or histological lesions [17, 21]. In sheep, *C. psittaci* infection has been associated with destruction of the chorionic epithelium [45], but the mechanism of abortion in mares is unknown [17]. Further work is needed to determine the mechanisms by which *C. psittaci* may induce abortion in mares.

In this study we used *ompA* PCR and sequencing to successfully genotype 17 of the 39 postive cases. Genotyping cannot always be performed directly on clinical samples due to low concentrations of DNA [46], which may explain why genotyping was not successful in the remaining positive samples. Molecular analysis of *C. psittaci* detected in this study revealed that the samples clustered together in the pathogenic genotype A/6BC clade, corresponding to ST24. The 6BC/ST24 *C. psittaci* genotype has mainly been detected in parrots, including wild and captive psittacines in VIC [32, 47]. All cases of *C. psittaci* equine abortion cases in Australia have been the 6BC/ST24 genotype [21, 23, 24, 26]. These results suggest that Australian parrots may be the reservoir of equine *C. psittaci* infection in Australia [21, 24, 26]. Whether *C. psittaci* infection in horses occurs only by sporadic spillover from parrots or whether this pathogen can develop stable infection in horse populations remains to be determined.

The detection of the highly virulent 6BC clade of *C. psittaci* in equine abortion cases in Australia is of considerable public health importance. A cluster of human *C. psittaci* infections has been reported following exposure to equine foetal membranes within an Australian veterinary school [12, 21]. Future measures to decrease the risk of human infection are recommended, including the use of appropriate personal protective equipment and biosecurity procedures. Such measures may also assist in preventing spread of infection between horses. Surveillance for the detection of *C. psittaci* in horses is recommended, as is routine diagnostic testing for *C. psittaci* in cases of equine abortion.

## Supplementary Data

Supplementary material 1
